# Portable Multichannel Measurement System for Real-Time Microplastics Assessment Using Microwave Sensors

**DOI:** 10.3390/s26020669

**Published:** 2026-01-19

**Authors:** André Barrancos, Diogo Rosinha, Jorge Assis, Luís S. Rosado

**Affiliations:** 1 Instituto de Telecomunicações, Av. Rovisco Pais 1, 1049-001 Lisbon, Portugal; andre.barrancos@tecnico.ulisboa.pt (A.B.);; 2 Instituto Superior Técnico, University of Lisbon, Av. Rovisco Pais 1, 1049-001 Lisbon, Portugal; jorge.assis@tecnico.ulisboa.pt

**Keywords:** microwave sensor, measurement system, multichannel, microplastics

## Abstract

This paper presents a multichannel electronics measurement system that uses microwave sensors to perform real-time microplastics assessment in aqueous environments. The system is capable of simultaneously reading up to four microwave sensors, enabling the use of multiple sensors that target microplastic particles with different sizes and properties. The multichannel capability allows the measurement of multiple MW sensors integrated with different microfluidic channel designs while targeting different MPs’ dimension ranges, although experimental validation in this work was limited to a single sensor. Each readout channel is implemented combining radio-technology-integrated circuits with a microprocessor that has advanced analog peripherals used for signal conditioning and acquisition. An ADF4351 wideband frequency synthesizer is used for excitation signal generation while an ADL5902 power detector converts the sensor output to a DC voltage. Baseline removal and amplification of the power detector output is realized with a MSP430FR2355 microprocessor which is also responsible for its acquisition at 40 kHz and digital decimation. Characterization results show the system’s capability to generate excitation signals between 700 MHz and 3.5 GHz with power levels around 0 dBm. Sensor output can be detected with a power between −50 dBm and −5 dBm and a 230 Hz bandwidth. A compact form factor of 15 cm × 10 cm × 3 cm was realized together with a low power consumption of 6.6 W. Validation was realized with a previously developed microwave sensor, demonstrating the detection of polyethylene spheres with 400 μm diameters animated in 10 mL/min flux within the microfluidics device.

## 1. Introduction

Plastic is a widespread material in our modern society due to its affordability, durability and versatility [1]. Major issues arise when plastic ends up in the environment, which, due to solar exposure, wind and other natural factors, breaks down into increasingly smaller particles and eventually becomes microplastics (MPs) once their size is smaller than 5 mm [2]. MPs are synthetic and non-biodegradable, leading to their continuous accumulation in the environment as their degradation may take several decades. This leads to an increase in MPs’ presence in oceans, drinking water, seafood and even human organs. Recent studies suggest that, on average, humans ingest between 0.1 g and 5 g of MPs per week [3]. Part of this contamination comes from tap water, with MP concentration typically at 3.76 particles per liter [4], while bottled water has been found to be contaminated with 63.9 MP particles per liter [5]. Despite the exponential increase each year of MP pollution, there is a remarkable research gap in how MPs affect human health and our ecosystems. Thus, it is essential to develop systems that can accurately measure contamination levels, especially in aqueous environments [6].

Currently, most MP sample analyses are performed in laboratorial conditions using techniques like microscopy [7], Fourier Transform Infrared spectroscopy (FT-IR) [8], Raman spectroscopy [9] and pyrolysis gas chromatography–mass spectrometry (PY-GC-MS) [10]. Despite being effective, these methods are not suited for continuous monitoring as sample preparation is barely automatized while the required instrumentation is bulky, requires a high amount of power to operate and is very expensive.

Recent developments in research have culminated in new real-time monitoring systems to assess the number of MPs in aqueous environments. These systems enable the collection of crucial real-time data to detect pollution events and provide a better understanding of MP sources and dissemination. Portability, energy-efficiency and affordability are key targets that need to be met in order for these systems to be widely deployed [6].

MW sensors have been used to assess different dielectric materials and properties [11]. Examples include the food industry, where these sensors have been used to detect metallic contaminants in dry food [12], and also in non-destructive testing, where they are used to look for the smallest imperfections in critical mechanical components [13].

These sensors have equally emerged as a promising solution for water quality monitoring. Recent studies have shown their capability for detecting bacteria [14], toxic metals [15] and MPs [16]. Concerning MP assessment, MW sensors were demonstrated measuring bulk concentrations as low as 100 ppm [17,18]. The integration of such sensors with microfluidics devices has enabled continuous and real-time MP assessment. For instance, small particles with sizes down to 20 μm were detected using compact and cost-effective MW sensors in [19,20].

Despite these appealing MW sensor characteristics, they are mostly paired with Vector Network Analyzers (VNAs) which are far from suitable for field deployment. Thus, some developments have already been reported for portable readout systems, such as in [21], where a simple and straightforward approach was presented using a voltage-controlled oscillator (VCO) and analog detectors for permittivity imaging. Also, for MP detection, System-On-Chip (SOC) solutions for environmental monitoring have been proposed. In both [22,23], a SOC system was developed to measure bulk MP concentration, showing the potential of these approaches for compact and portable MP monitoring systems. To the best of our knowledge, there are no readout systems suitable for in situ, continuous and real-time MPs monitoring.

This paper introduces a new multichannel electronics measurement system able to read different and multiple MW sensors, granting a compact/portable form factor, energy efficiency and cost effectiveness. The proposed system was designed for modularity by relying on several MW measurement sub-system instances that can be populated depending on the sensors on hand. All combined, these features make this innovative and advanced system a valuable measurement solution to pair with MW sensors applied for in situ, continuous and real-time MPs assessment.

## 2. Theoretical Background

**Figure 1:**
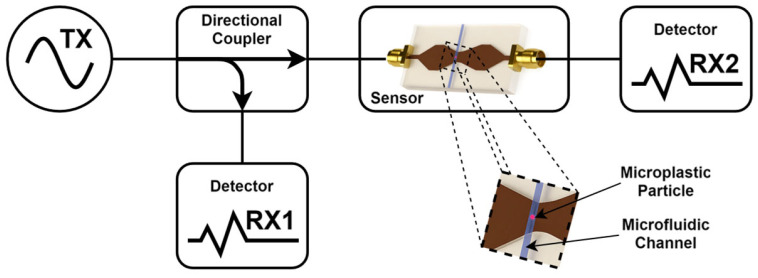
Generic reflection and transmission MW sensors and measurement electronics. The MW sensor is integrated with a microfluidics device where water and MPs flow across.

An example of a generic reflection and transmission MW sensor paired with measurement electronics is shown in Figure 1. The MW sensor includes an electromagnetic structure designed to promote electric field interaction with MPs that are present in the microfluidic water flow. A signal generator (TX) provides an excitation signal used to energize the sensor input port. MPs may be detected through measuring the reflection coefficient $S_{11}$ at the sensor input port. For this, directional couplers are used to sample the reflected energy, later measured by a detector (RX1). For some sensors, MP detection be equally achieved through measuring the transmission coefficient $S_{21}$ using a detector (RX2) at the sensor output port. Eventually, when the sensor allows, the combination of both measurements is expected to provide better results.

**Figure 2:**
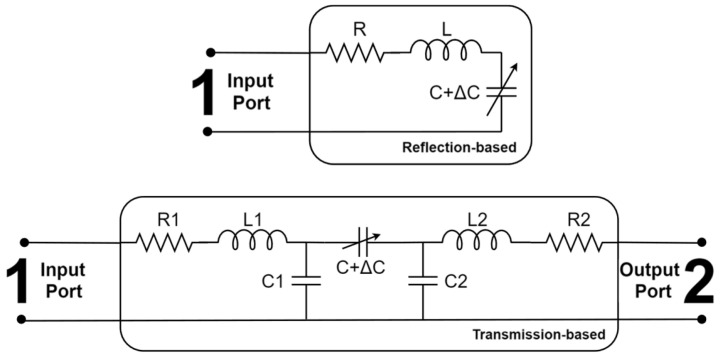
MW sensor’s equivalent circuits.

Usually, MW sensors are designed with resonant electromagnetic structures offering a high-quality factor (Q Factor). Significant research has been carried out to explore different MW structures types, including coplanar waveguides (CPW) [24], split-ring resonators (SRR) [19] and microstrips [25]. Usually, these structures have some kind of gap or discontinuity which becomes the MPs sensitive zone and where the microfluidic channel is processed. The microfluidic device has the important task of guiding the water and MPs through a usually small sensitive zone, improving the sensor response when the MPs interact evanescent electric field. Commonly, RLC circuits are used to model the sensor electrical behavior; see Figure 2.

Capacitance changes, ΔC, are influenced by the presence of MPs in the sensitive zone, which changes the MW sensors’ overall impedance and affects the reflected or transmitted power levels at the input and output ports, respectively. In some cases, measurement is performed by tracking the resonance frequency or, in other scenarios, with a fixed frequency near the resonance. For both cases, the amplitude and phases of the signals hold information on the MP particles.

## 3. Measurement Hardware

**Figure 3:**
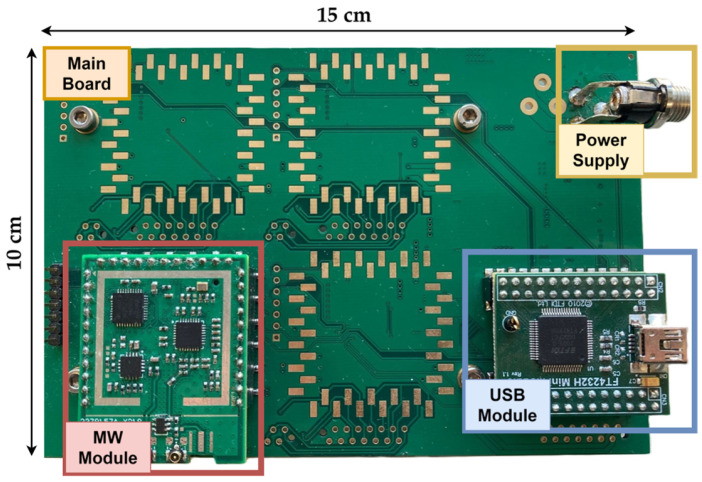
Developed MW measurement system.

The developed system includes a motherboard where several MW measurement sub-systems are populated through pin header connectors; see Figure 3.

### 3.1. Motherboard

**Figure 4:**
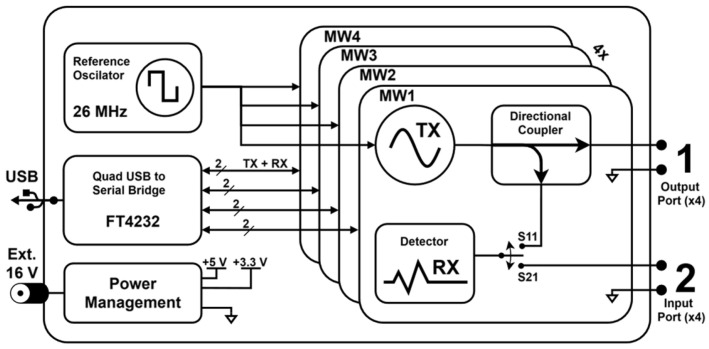
Main board block diagram.

The system’s main board is responsible for providing the necessary power supplies, clock references and communication interfaces for the four MW measurement sub-systems that can be included in the system; see Figure 4.

External power is provided by an external mains adapter with a +16 V output. A DC-DC converter TPS5430 steps this voltage down to +12 V and a linear regulator LM3940 is used to generate the +5 V needed by the selected MW power detector. An additional LM3940 generates the +3.3 V needed for the excitation signal generation and the microprocessors in the MW measurement sub-systems. The overall system has a power consumption of 6.6 W with a current of 412 mA. The power management unit was designed with the consideration of the future integration of a Jetson Nano processing core. This option resulted in higher power consumption due to the inefficiency of the chosen regulators under the light load conditions without the processing core.

A 26 MHz buffered oscillator SG7050CA24.0TJGA3 is used as the clock reference for all MW measurement sub-systems. This design decision enables the synchronization of several signal generators and the avoidance of frequency misalignment interferences across the sub-systems. Additionally, it also enables the generation of excitation signals with precise phase or frequency offsets.

Communication between the four MW measurement sub-systems and a computer is achieved through USB-emulated serial ports using the FTDI FT4232 mini-module. This high-speed USB-UART bridge allows bitstreams up to 640 kbit/s, which is enough for the bitstream generated by the four sub-system instances.

### 3.2. MW Module

**Figure 5:**
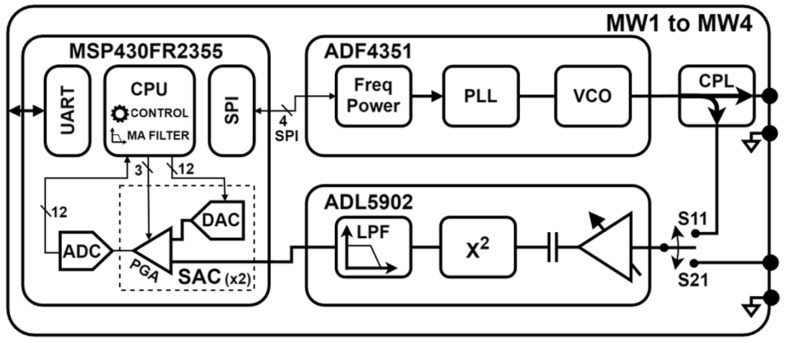
MW measurement sub-system block diagram.

The overall architecture of an MW measurement sub-system is shown in Figure 5.

The MW excitation signal generator was designed using ADF4351 (Analog Devices, Wilmington, MA, USA) [26], a wideband frequency synthesizer that integrates a voltage-controlled oscillator (VCO), a phase-locked loop (PLL) and multiple output frequency dividers, with this integrated circuit operating between 35 MHz and 4.4 GHz. Output power can be adjusted within four different levels with 3 dB steps.

For reflection-based sensors, the power reflected at the input port is sampled using a D17W+ [27] directional coupler, which grants effective isolation at an acceptable insertion loss. This coupler was designed for operation between 700 MHz and 3.5 GHz. When measuring transmission-based MW sensors, the directional coupler can be bypassed with a minor physical passive component modification.

The reflected or transmitted power is finally measured by ADL5902 (Analog Devices, Wilmington, MA, USA) [28], a high-precision true Root Mean-Square (RMS) power detector that operates between 50 MHz and 9 GHz. This integrated circuit generates a DC continuous voltage proportional to the input RMS. Conversion gain is governed by external passive components being configured to 46.2 mV/dBm.

The power detector output connects to an MSP430FR2355 microcontroller (Texas Instruments, Dallas, TX, USA) [29] which includes two Smart Analog Combo (SAC) and one Analog-to-Digital Converter (ADC). The two SACs are set in cascading mode [30], allowing amplification gains up to 1024, while the DACs are used to generate reference voltages for baseline removal. This baseline removal and amplification is essential to deal with the low signal amplitude paired with the high baseline voltage at the power detector output. Overall, this design enables a better exploration of the ADC dynamic range. The ADC is configured to produce 40 kSamples/s which are later processed by the microcontroller’s CPU, where a moving average is used to filter before decimation.

## 4. Measurement System Firmware/Software

### 4.1. Firmware

The firmware developed for the MSP430FR2355 manages the necessary configurations and signal acquisition/processing. The firmware allows the enablement/disablement and adjustment of the excitation signal generator (power and frequency); the adjustment of the PGA gains; the changing of the baseline compensation value at the DAC output; the changing of the ADC acquisition rate; and the changing of the moving average filter integration length.

Commands are exchanged with a Graphical User Interface (GUI) using a proprietary protocol inspired by High-Level Data Link Control (HDLC). Only the strictly necessary functionalities were preserved like frame delimiters, byte stuffing and error detection.

### 4.2. Graphical User Interface

**Figure 6:**
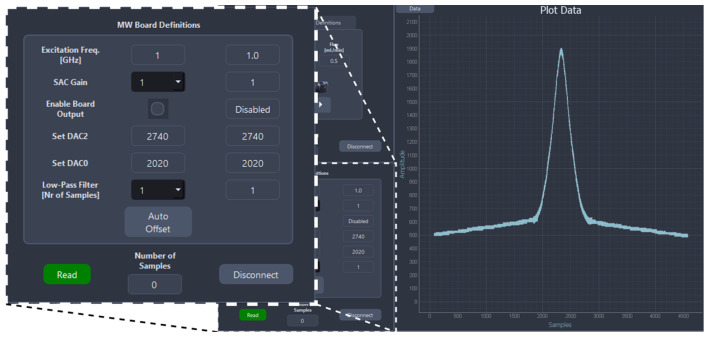
GUI’s front panel. MW measurement configuration sub-panel (close-up, (**left**)) and MP particle detection result plot (main panel, (**right**)) are shown.

The front panel for the developed system is shown in Figure 6. It enables the configuration of the operational parameters of the several MW measurement sub-systems. An additional button, “Auto Compensation”, can trigger the compensation algorithm, which automatically configures the baseline compensation values at the DAC output, meaning that the input of the ADC is brought near mid-range, i.e., 1.25 V.

Besides the functionalities to control the MW sub-systems, the GUI allows the user to control microfluidic pumps, specifying the desired volume to be dispensed and the flow rate. In addition to all the control functionalities, the GUI enables the visualization of the data in real-time, plotting the data with a 5.5 kHz sampling rate. Compared with the VNA setup used in [31], which had a sampling rate of only 100 Hz, the proposed setup provides a substantial improvement, which is needed to achieve 10 mL/min inlet flow rate while maintaining real-time capability. Finally, the results are saved in a MATLAB 2024a file format for further analysis.

## 5. Experimental Characterization

The MW measurement sub-system was experimentally characterized regarding stability, noise and bandwidth. To perform these tests, the MW sub-system directional coupler was bypassed, leaving individual U.FL connections to the signal generator ADF4351 and to the power detector ADL5902.

**Figure 7:**
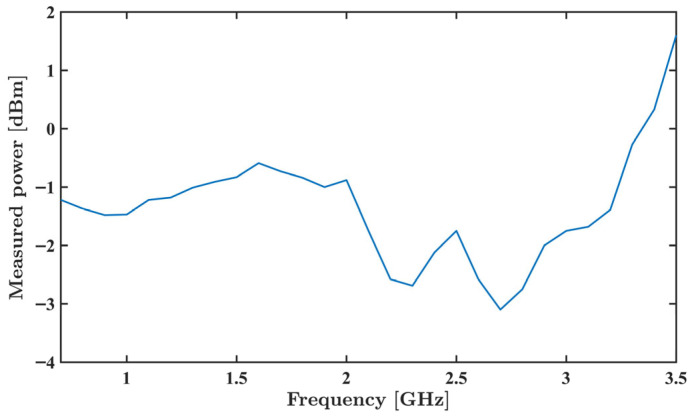
Acquired ADF4351 measured output power as a function of frequency between 700 MHz and 3.5 GHz.

The excitation signal generator was characterized using a RSA3045N spectrum analyzer (Rigol Technologies, Suzhou, China) while successively programming output frequencies between 700 MHz and 3.5 GHz; see Figure 7. These frequencies were selected because, within this frequency range, the water’s permittivity is much higher than the MPs’, with the higher permittivity contrast allowing MP detection [32]. Also, at these frequencies, the electric field penetrates the MP particles, making it possible to measure their permittivity [33].

Analyzing Figure 7, a substantially flat response is observed up to 2 GHz. After this value, the PCB layout limitations and potential manufacturing deviations introduced substantial changes across the frequencies.

A E4438C Vector Signal Generator (VSG) (Agilent Technologies, Santa Rosa, CA, USA) was connected to the power detector. In the first analysis, the VSG was set to generate a −10 dBm continuous wave while varying the frequency between 700 MHz and 3.5 GHz, with a 100 MHz step. In the second experiment, the power was set from −50 dBm to −10 dBm, with −5 dBm steps, with a fixed 3 GHz frequency. The gain at the MSP430FR2355 SACs was set to the maximum possible while the DACs were adjusted to bring the ADC near mid-range.

**Figure 8:**
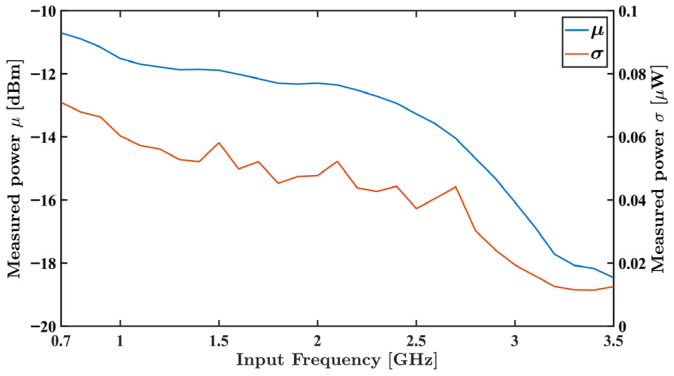
Acquired voltage average (blue) and standard deviation (orange) as functions of frequencies between 700 MHz and 3.5 GHz with a fixed power input of −10 dBm.

**Figure 9:**
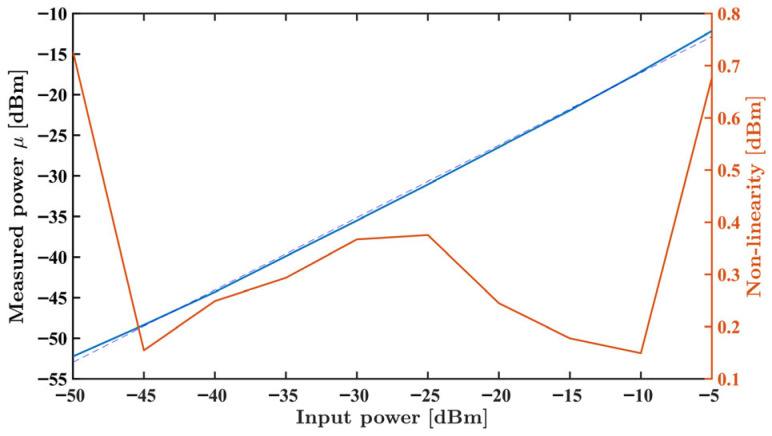
Measured power mean value (blue) and non-linearity (orange) for input powers ranging from −50 dBm to −5 dBm at a fixed frequency of 3 GHz.

For each test, one million samples were acquired using the MSP430FR2355 microcontroller. The measured results are presented in Figure 8 and Figure 9, showing the measured average output voltage, in blue, and the standard deviation, in orange, as functions of frequency and power, respectively.

In both results, minimal variation in the standard deviation for the overall data is observed, attesting the system stability across frequency and input power. As expected from the ADL5902 datasheet [28], its sensitivity decreases with growing frequency, as observed in Figure 8. As also expected from the manufacturer data, a highly linear dB sensitivity can be observed in Figure 9. The maximum non-linearity of 0.73 dBm, 9.2%, is observed at −50 dBm input power.

**Figure 10:**
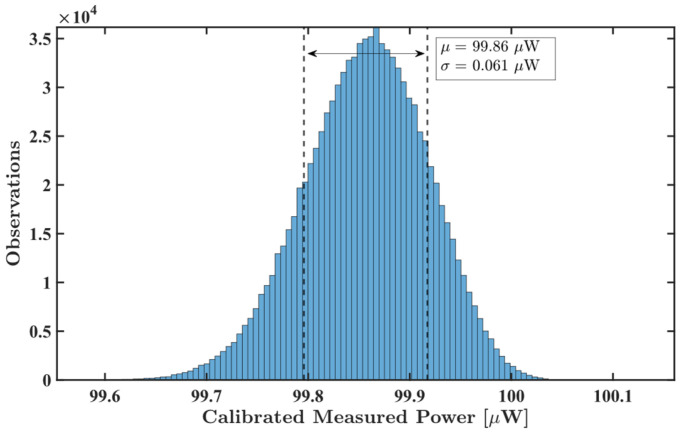
Histogram of the acquired samples with a −10 dBm, 100 µW input signal at a 3 GHz frequency.

**Figure 11:**
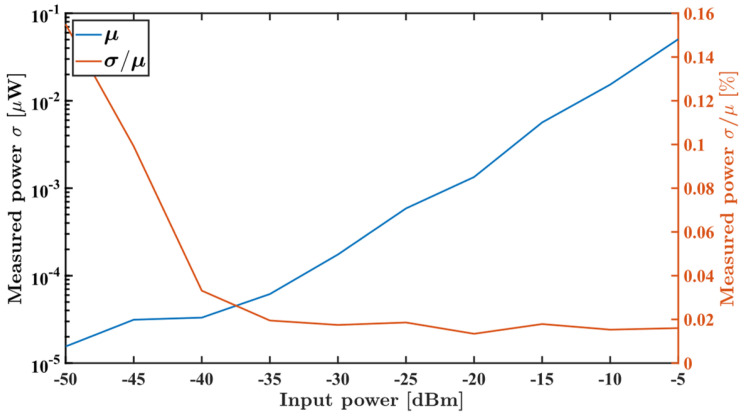
Measured power standard deviation (blue) and relative standard uncertainty (orange) for input powers ranging from −50 dBm to −5 dBm at a fixed frequency of 3 GHz.

Figure 10 shows a histogram of the one million samples acquired at −10 dBm at a frequency of 3 GHz, displaying the average measured value and standard deviation for those samples. The same was performed for samples acquired with different input powers; Figure 11 reveals a relative standard uncertainty around 0.16% at 50 dBm and below 0.02% for an input power greater than 35 dBm.

The next test was used to determine the developed measurement system’s bandwidth. The E4438C VSG was used to produce an amplitude-modulated (AM) sinusoidal signal with a central frequency of 3 GHz that was applied to the power detector’s input. The modulated sinewave was set to swing between −40 dBm and −10 dBm while its frequency swept between 0.1 Hz and 10 kHz.

**Figure 12:**
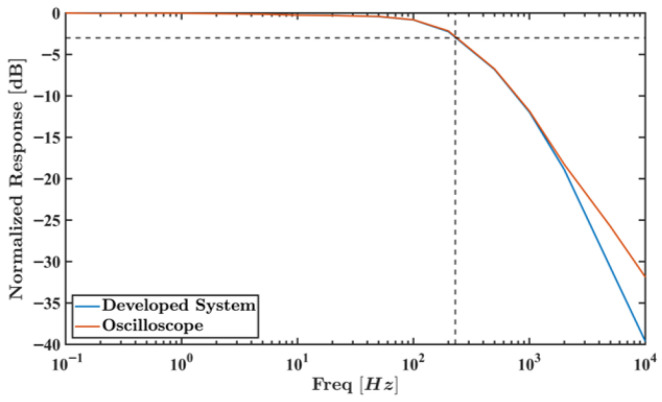
Developed measurement system’s frequency response to an AM sine waveform at a 3 GHz central frequency swinging between −40 dBm and −10 dBm. Normalized amplitudes computed from the MSP430FR2355 acquisitions (blue) and from the oscilloscope (orange) are shown.

For each frequency level, a total of 1,000,000 samples were acquired by the MSP430FR2355 microcontroller. The amplitude of the fundamental was computed from the acquired samples’ DFT (while considering the spectral leakage effects). As a cross-check, the output of the power detector was digitally acquired using a Tektronix TBS2000 Series oscilloscope (Tektronix , Beaverton, OR, USA) and similarly processed. Figure 12 shows the bandwidth characterization results between 0.1 Hz to 10 kHz. Both plots confirm an overall bandwidth of 230 Hz, which matches the ADL5902 passive component dimensioning.

## 6. Experimental Validation

**Figure 13:**
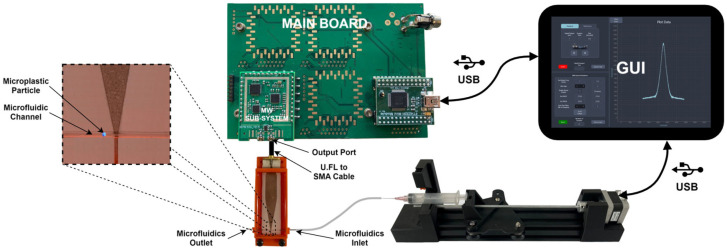
MPs assessment pilot installation, including the developed MW measurement system.

The developed measurement system was integrated within a pilot installation for its validation; see Figure 13. This pilot installation includes an MW sensor, originally proposed in [25] and enhanced in [31], and a microfluidic pump. The pump was adapted from [34] and is used to pump water through the sensor’s microfluidic channel and move a polyethylene MPs sphere that is 400 μm in diameter.

Initially, the MW sensor’s resonant frequency was measured at 3.1 GHz with a RSA3045N spectrum analyzer with VNA capabilities. Afterwards, the MW sensor was connected to the measurement system; as the sensor operation is reflection based, the directional coupler was included to sample the power reflected in the input port. The system signal generator was set to the maximum, a frequency of 3.1 GHz. The MSP430FR2355 SACs gains were set to 16 and the digital low pass filter to a 1.25 kHz cut-off frequency.

**Figure 14:**
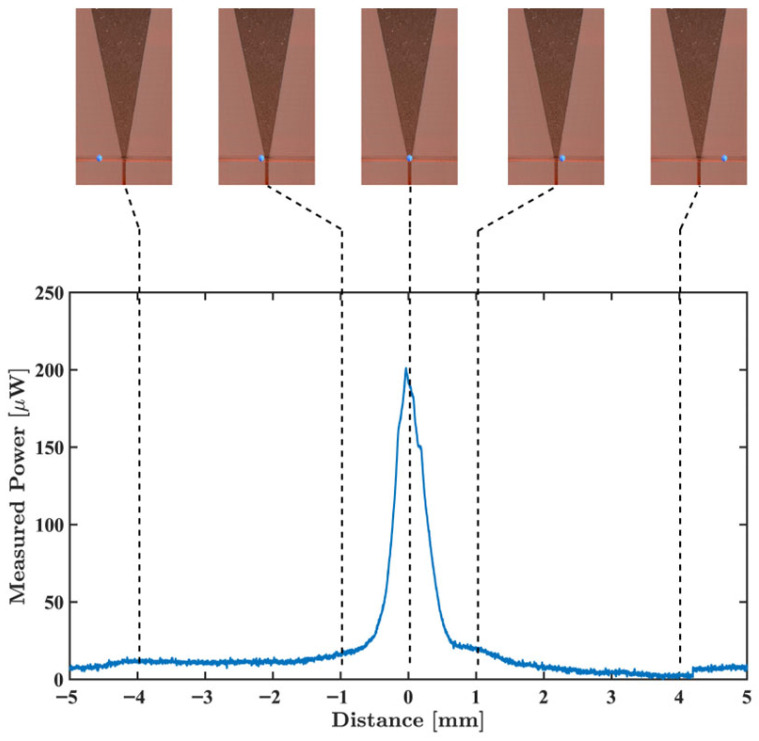
MW sensor’s response for each successive position of the polyethylene MP sphere—calibrated values. The upper images show the relative positioning between the sphere and the sensor sensitive zone.

Finally, the sensor response was measured while the polyethylene MP sphere ($\varepsilon_{r}=2.3,\tan\left(\delta \right)=0.0004$) was moved across the microfluidic channel with a steady flux of 10 mL/min; see Figure 14. The programmed flow rate was set to a maximum of 10 mL/min, considering the sensor characteristics and the detector’s bandwidth. The employed sensor response ranges around 2 mm along the microfluidics channel, as demonstrated in [31]. Considering the detector’s bandwidth of 230 Hz, and that the MPs detection may accept the loss of high-frequency response features, a particle has a total of Δt = 4.38 ms to travel across the 2 mm-long sensitive zone. Based on the inner diameter of the microfluidic channel, the maximum linear speed and maximum inlet flow rate are around 460 mm/s and 10 mL/min, respectively.

Finally, the dimension of the smallest detectable particle by the demonstrated system can be calculated. It is known that the ΔC from Figure 2 is related to the particle radius, and, as the radius reduces, ΔC decreases cubically [35]. Also, since the reflected power variation that a MP particle produces is proportional to ΔC [19], one obtains

(1) $${\left(\frac{a^{\prime }}{a}\right)}^{3}=\frac{p^{\prime }}{p},$$

where a’ is the minimum detectable particle radius. Here, a is the 200 µm particle radius, while p is the reflected power by this MP particle (200 µW obtained from Figure 14) and p’ is the noise level of the developed system (0.061 µW obtained from the standard deviation shown in Figure 10). Taking both the previous condition and a 0 dB SNR condition, the radius of the smallest detectable particle is approximately 13 µm.

## 7. Conclusions

This paper presented an innovative multichannel electronics measurement system designed to read multiple and different types of MW sensors while answering requirements on the in situ, continuous and real-time MPs assessment. This ability of simultaneously measuring several sensors is important for the assessment of MPs.

Instead of conventional synchronous demodulation, the proposed architecture explores the use of MW power detectors. This choice is sustained by the near-field operation and high-quality factor verified in MW sensors designed for MPs, both contributing to minimal susceptibility to external interferences (resulting from RF devices operating in their vicinity). Characterization results showed the system’s stability across a 700 MHz to 3.5 GHz frequency range while measuring power levels between −50 dBm and −5 dBm. Analog bandwidth was determined at 230 Hz, which is high enough to accommodate the dynamics of the small MP particles traveling at usual microfluidic flow rates and channel dimensions.

The system was validated by detecting a polyethylene MP sphere with a 400 μm diameter flowing through the MW sensor’s microfluidic channel at 10 mL/min. The detected MP generated a signal response of around 200 μW. The MW system’s overall specifications are presented in Table 1.

Future work will focus on the integration of an NVIDIA Jetson Nano computing platform to achieve fully autonomous operation without relying on a personal computer. The final goal is to deploy the new system in a new pilot installation for the continuous detection and characterization of MPs, utilizing Machine Learning algorithms executed in the Jetson Nano.

## Figures and Tables

**Table 1:** Developed system’s general specifications.

Number of Channels	Up to 4
Frequency Range	700 MHz to 3.5 GHz
Baseline Compensation	12 bits each stage 1.61 mV each stage
Amplification Gain	Up to 32× each stage 1024× overall
Acquisition Sampling Rate	40 kSamples/s
Output Sampling Rate	5.5 kSamples/s
ADC Resolution	12 bits
Voltage Supply	16 V
Power Consumption	6.6 W
Overall Size	15 cm × 10 cm × 3 cm
Total Price	EUR < 200

## Data Availability

The data presented in this study is not publicly available at this time but may be obtained from the authors upon reasonable request.
